# DNA methylation in glioblastoma: impact on gene expression and clinical outcome

**DOI:** 10.1186/1471-2164-11-701

**Published:** 2010-12-14

**Authors:** Amandine Etcheverry, Marc Aubry, Marie de Tayrac, Elodie Vauleon, Rachel Boniface, Frederique Guenot, Stephan Saikali, Abderrahmane Hamlat, Laurent Riffaud, Philippe Menei, Veronique Quillien, Jean Mosser

**Affiliations:** 1 CNRS UMR6061 Institut de Génétique et Développement, Université de Rennes 1, UEB, IFR140, Rennes, France; 2 Service de Génétique Moléculaire et Génomique, CHU Rennes, France; 3 Plateforme Génomique Santé Biogenouest®, Rennes, France; 4 INSERM U946, Fondation Jean Dausset, CEPH, Paris, France; 5 Département de Biologie Médicale, Centre Eugène Marquis, Rennes, France; 6 Service d'Anatomie et Cytologie Pathologique, CHU Rennes, France; 7 Service de Neurochirurgie, CHU Rennes, France; 8 Service de Neurochirurgie, CHU Angers, France

## Abstract

**Background:**

Changes in promoter DNA methylation pattern of genes involved in key biological pathways have been reported in glioblastoma. Genome-wide assessments of DNA methylation levels are now required to decipher the epigenetic events involved in the aggressive phenotype of glioblastoma, and to guide new treatment strategies.

**Results:**

We performed a whole-genome integrative analysis of methylation and gene expression profiles in 40 newly diagnosed glioblastoma patients. We also screened for associations between the level of methylation of CpG sites and overall survival in a cohort of 50 patients uniformly treated by surgery, radiotherapy and chemotherapy with concomitant and adjuvant temozolomide (STUPP protocol). The methylation analysis identified 616 CpG sites differentially methylated between glioblastoma and control brain, a quarter of which was differentially expressed in a concordant way. Thirteen of the genes with concordant CpG sites displayed an inverse correlation between promoter methylation and expression level in glioblastomas: *B3GNT5*, *FABP7*, *ZNF217*, *BST2*, *OAS1*, *SLC13A5*, *GSTM5*, *ME1*, *UBXD3*, *TSPYL5*, *FAAH, C7orf13*, and *C3orf14*. Survival analysis identified six CpG sites associated with overall survival. *SOX10 *promoter methylation status (two CpG sites) stratified patients similarly to *MGMT *status, but with a higher Area Under the Curve (0.78 vs. 0.71, *p-*value < 5e-04). The methylation status of the *FNDC3B*, *TBX3*, *DGKI*, and *FSD1 *promoters identified patients with *MGMT*-methylated tumors that did not respond to STUPP treatment (*p-*value < 1e-04).

**Conclusions:**

This study provides the first genome-wide integrative analysis of DNA methylation and gene expression profiles obtained from the same GBM cohort. We also present a methylome-based survival analysis for one of the largest uniformly treated GBM cohort ever studied, for more than 27,000 CpG sites. We have identified genes whose expression may be tightly regulated by epigenetic mechanisms and markers that may guide treatment decisions.

## Background

Glioblastoma (GBM) is the most common and aggressive primary brain tumor in adults. Its prognosis remains extremely poor, despite multimodal treatment by surgery, radiotherapy and, chemotherapy [1]. These tumors are now well characterized at the transcriptome and genome levels. Several studies have demonstrated that a combination of these two molecular levels may be advantageous for determining robust signatures and clinically relevant molecular classifiers of GBM [2,3].

The role of general epigenetic mechanisms in carcinogenesis and tumor aggressiveness is well documented: CpG island hypermethylation silences tumor suppressor genes, whereas hypomethylation promotes the transcriptional activation of oncogenes and induces chromosomal instability [4,5]. Such epigenetic changes are potentially reversible and may therefore be considered promising targets for epigenetic anticancer treatments. Indeed, the use of DNA-demethylating drugs (5 azacytidine and 5-aza-2'-deoxicytidine) has been approved by the Food and Drug Administration (FDA) as a treatment for myelodysplastic syndromes and myelogenous leukemia [6,7].

Changes in promoter DNA methylation pattern of genes involved in key biological pathways have been reported in GBM. For instance, the retinoblastoma (RB), PI3K, and p53 pathways are affected by CpG island promoter hyper-methylation (*RB*, *CDKN2A*, *PTEN*, *TP53*) [8-12]. Epigenetic silencing of the *O^6^-methylguanine DNA methyltransferase *(*MGMT*) gene, which encodes a DNA repair enzyme, sensitizes cancer cells to alkylating agents, and is associated with significantly longer survival in GBM patients treated by radiotherapy and concomitant and adjuvant temozolomide [13]. According to the European Organization for Research and Treatment of Cancer (EORTC) and the National Cancer Institute of Canada (NCIC) trial 26981-22981/CE.3, the methylation status of the *MGMT *promoter is the strongest predictor of outcome and benefit from temozolomide treatment [14].

An instructive mechanism for *de novo *methylation has also been described in cancers [15]. This mechanism involves polycomb group proteins known to repress genes epigenetically at the embryonic stem cell (ESC) stage. Indeed, recent studies have shown that the polycomb repressor complex 2 (PRC2) may mark genes repressed during the ESC stage and induce their targeted silencing in cancer [16].

Genome-wide assessments of DNA methylation are now necessary, to decipher the epigenetic events involved in the aggressive phenotype of GBM and to guide new treatment strategies. Several microarray-based GBM studies have identified gene promoters that are frequently hyper- and hypomethylated. These gene promoters were initially identified indirectly, by the pharmacologic or RNAi-induced inhibition of DNA methyltransferase in GBM cell lines [17,18], or by the use of methyl-CpG-binding proteins [19]. More recently, direct hybridization of bisulfite-modified DNA on beadchips has made it possible to reliably quantify promoter methylation [20,21] in cohorts of patients. Noushmehr *et al*. used this technique to profile DNA methylation alterations in 272 GBMs in the context of The Cancer Genome Atlas (TCGA). They reported a rare subgroup of GBMs displaying a concerted multilocus hypermethylation pattern and suggested the existence of a Glioma CpG Island Methylator Phenotype (G-CIMP). G-CIMP tumors tended to be secondary and recurrent GBMs, and were tightly associated with *IDH1 *somatic mutation.

We report here the first genome-wide integrative analysis of DNA methylation and gene expression profiles obtained from the same GBM cohort. We also present a methylome-based survival analysis for one of the largest uniformly treated (radiotherapy and chemotherapy with concomitant and adjuvant temozolomide) GBM cohort ever studied, for more than 27,000 CpG sites. We identified frequent tumor-specific methylation changes in GBM. Some of these alterations directly affected gene expression, whereas others were significantly associated with the clinical outcome of patients.

## Methods

### Tissue samples

The prospective cohort included 55 patients with newly diagnosed GBM (World Health Organization (WHO) grade IV), admitted to the Neurosurgery Departments of Rennes and Angers University Hospitals. Tumor samples were collected, following informed consent, in accordance with the French regulations and the Helsinki Declaration. Initial histologic findings were confirmed, according to the WHO classification [22], by a central review panel including at least two neuropathologists. The male/female ratio was 1:0.96. Median age at diagnosis was 57.5 ± 12 years (range: 26 - 80 years) and median preoperative Karnofsky Performance Status (KPS) was 78.6 (range: 40 - 100). Fifty patients underwent radiotherapy and chemotherapy with concomitant and adjuvant temozolomide (STUPP protocol). Four patients received only fractionated radiotherapy (60 Gy). One patient died after surgery. Median overall survival (OS) was 18.7 ± 17.3 months (range: 0.2 - 98.6 months). Five non-neoplastic brain tissues obtained from patients undergoing surgery for chronic epilepsy were included in the study as control samples. Each snap-frozen tumor block was cut into 10 μm sections. For accurate paired comparisons between biological materials, adjacent sections were used for DNA and RNA extraction. We investigated the expression profiles of 40 GBMs for which methylation data were also available.

### DNA and RNA isolation

DNA was extracted with the NucleoSpin Tissue Kit (Macherey Nagel) according to the manufacturer's instructions. The quality of DNA samples was assessed by electrophoresis in a 1% agarose gel. Total RNA was isolated with the NucleoSpin RNAII Kit (Macherey-Nagel). RNA integrity (RNA Integrity Number ≥ 8) was confirmed with an Agilent 2100 Bioanalyzer (Agilent Technologies).

### DNA methylation profiling

DNA methylation profiling was performed with the Infinium HumanMethylation27 beadchip (Illumina Inc.), which interrogates 27,578 highly informative CpG sites located within the proximal promoter regions of 14,475 genes (1,126 cancer-related genes). Nearly 73% of these CpGs were localized within CpG islands. DNA from GBMs and control brains were bisulfite-modified, using the EZ DNA methylation kit (Zymo Research) and hybridized according to the manufacturer's instructions. The profiling was performed on 55 GBMs and 3 non-neoplastic brains. We performed two intra- and inter-array replicates, the first one on a GBM sample and the other one on a non-neoplastic brain sample. The observed correlations between replicate samples (r > 0.99) demonstrate the high reproducibility of the technique. For each interrogated CpG site, methylation status is calculated by dividing the signal from the methylated probe (M) by the sum of signals for both methylated and unmethylated (U) probes (*GenomeStudio 2008.1*, Illumina Inc.): β = Max(M,0)/[Max(M,0) + Max(U,0) + 100]. This β-value provides a continuous and quantitative measurement of DNA methylation, ranging from 0 (completely unmethylated) to 1 (completely methylated). Missing values were imputed by nearest neighbor averaging (*impute *R package). DNA methylation values followed a non symmetric bimodal distribution (Additional file 1) and CpG sites were globally hypomethylated in both GBM and control brain samples (median β-value = 0.1). DNA methylation data have been submitted to Gene Expression Omnibus (GEO) repository under accession number "GSE22867".

### Determination of methylation thresholds on the basis of expression values

CpG probes were binned according to their β-values (windows 0.05 wide). For each bin, the maximum expression values of the genes corresponding to the CpG probes were averaged for all patients (n = 40).

### Differentially methylated (DM) CpG sites

Prior selection of the CpG sites displaying the highest DNA methylation variation was carried out, based on the standard deviation (SD ≥ 0.1). β-values were compared between GBMs and control brain tissues with Student t-tests with a Welch approximation. Adjusted *p*-values were calculated by controlling for the false discovery rate (FDR) with the Benjamini & Hochberg (BH) procedure (*multtest*, R package). CpG sites were considered significantly differentially methylated if the adjusted *p*-value was below 0.01 and the difference in β-values (Δβ GBM vs. control brain) was greater than 0.2.

### Pyrosequencing analysis

*MGMT *promoter pyrosequencing was performed with the PyroMark Q96 CpG MGMT kit (Qiagen), according to the manufacturer's protocol. The values obtained were averaged over the five CpG loci tested.

### Gene expression profiling

This study was performed on 40 GBM samples with 3 non-neoplastic brains as controls. Gene expression profiling was carried out with the Agilent Whole Human Genome 4 × 44 K Microarray Kit (Agilent Technologies). Total RNA was extracted, labeled and hybridized according to the kit manufacturer's recommendations. Data were log2-transformed and normalized (quantile normalization and baseline transformation) with *GeneSpring GX *software (Agilent Technologies). Gene expression data have been submitted to Gene Expression Omnibus (GEO) repository under accession number "GSE22866".

### Differentially expressed (DE) genes

We used a non-parametric rank product method to account for hybridization bias and to identify genes up- or downregulated in GBM vs. control brains (*RankProd *R package). Genes were considered significantly differentially expressed if the FDR was below 0.05 and the absolute fold-change (GBM vs. control brain) was greater than 2. A list of DE genes with absolute fold-change greater than 4 is provided in Additional file 2.

### Correlation analysis

This analysis was performed on 40 GBM samples with methylation and expression data available. Methylation and expression probes were paired on the basis of Entrez Gene ID concordance. We assessed the association between CpG site methylation and the level of expression of the corresponding genes, by calculating Pearson's correlation coefficient (r). The level of gene expression was considered to be inversely correlated with CpG site methylation level if the r value obtained was less than -0.5 and the *p*-value was less than 0.001.

### Survival analysis

Survival analyses were carried out on 50 patients who had undergone surgery, radiotherapy, and chemotherapy with concomitant and adjuvant temozolomide. We performed univariate Cox regression analyses on the CpG sites displaying the greatest variation of DNA methylation (SD > 0.15). β-values were used as the predictor and OS time (in months) was used as the response. CpG sites with a *p*-value lower than 0.05 were selected for further analysis. For each CpG site, the β-value threshold giving the best stratification *p*-value according to the log-rank test was selected for the identification of patients displaying hypomethylation (β-value ≤ threshold) and hypermethylation (β-value > threshold). Only CpG sites with a *p*-value below 0.001 were investigated further. Survival probabilities at 18 months, corresponding to the median OS in our cohort, were determined with a classical Cox model. Time-dependent ROC curve analyses were used to determine the area under the curve (AUC) for each CpG. All tests were stratified for the age of patients (above or below the age of 50 years). Analyses were carried out with the *survival *and *survivalROC *packages of R software.

### IDH1 mutation

The genomic region spanning wild-type R132 of IDH1 was analyzed by direct sequencing as previously described [23].

## Results

### Selection of CpG probes with direct effect on gene expression

**Figure 1:**
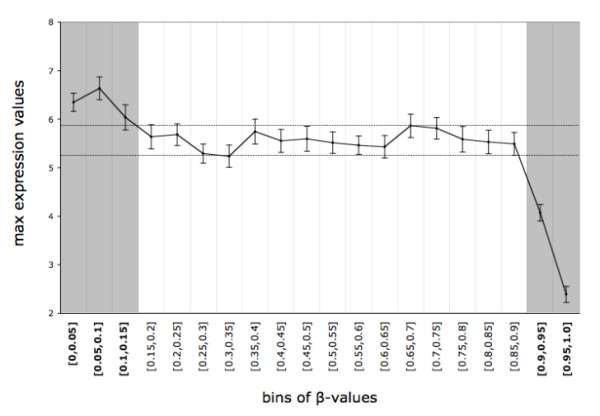
**Mean of the maximal gene expression values by β-value bins (5% wide), in GBMs (n = 40)**. The expression values presented are normalized and log-transformed intensities. Errors bars are also shown. Gray rectangles define the β-value ranges for which a change in maximal expression values is observed.

Expression levels remained almost constant for a broad range of β-values but the distributions were different for extremely low and high methylation values (Figure 1). We therefore identified CpG sites with a putative effect on gene expression levels as those with β-values below 0.15 or above 0.9 in at least three samples. This selection method led to the identification of 19,837 CpG sites (located within the promoter of 11,855 genes) and was used for DNA methylation profiling and correlation analysis.

### DNA methylation profiling of GBMs

**Figure 2:**
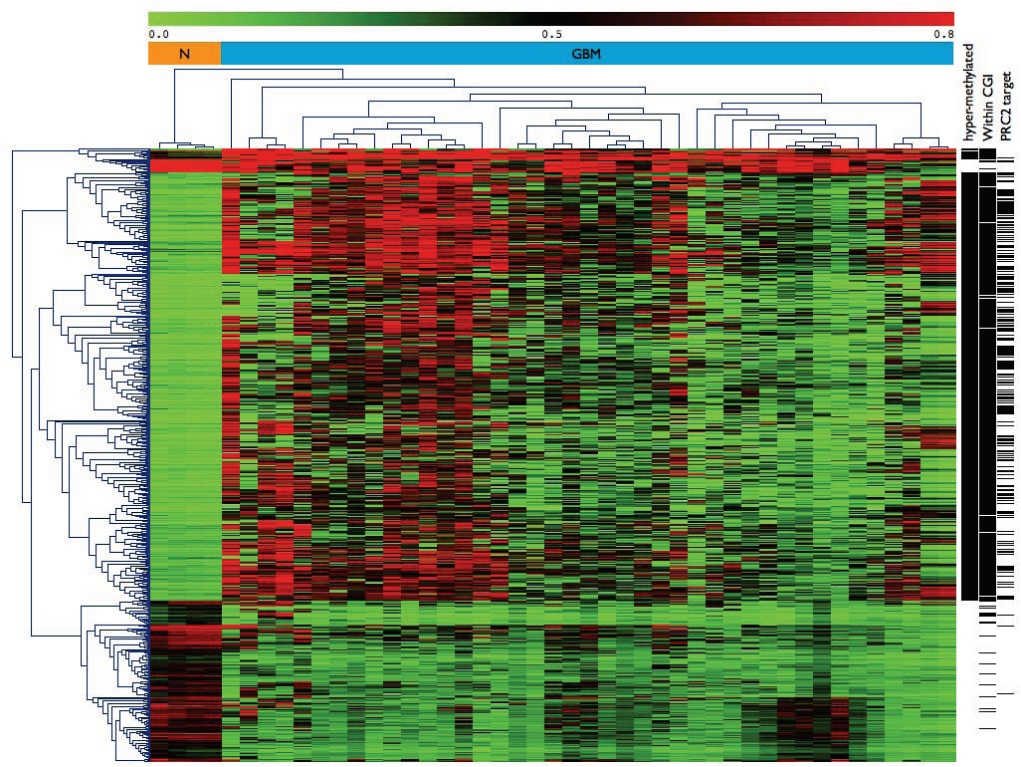
**Hierarchical clustering of the 616DM CpG sites in GBMs vs**. control brain (N). For each CpG site, a horizontal black bar on the right indicates membership of the hypermethylated subset, a CpG island (CGI), or the location within the promoter of a PRC2 target.

We found that 616 of the 4,344 selected CpG sites (SD ≥ 0.10) were DM between GBM and control brain samples: 440 CpG sites (358 genes) were hypermethylated and 176 (170) were hypomethylated in GBM (Additional file 3). Some of the identified changes in gene methylation have been reported before: the hypermethylation of *CDKN2A *(*p14ARF *and *p16ΙNK4a*) has been implicated in carcinogenesis and tumor progression [10], whereas the hypomethylation of *S100A2 *[24] has been identified as a strong inducer of metastasis *in vivo *in non small cell lung cancer [25]. As expected, unsupervised hierarchical clustering of the DM CpG sites clustered the samples into two distinct groups: the GBM samples and the control brain samples (Figure 2). CpG sites methylation patterns differed considerably between GBM patients. This heterogeneity was even more marked if we considered the hypermethylated CpG subset. This analysis also showed that some GBM samples were more strongly altered than others and we observed three main GBM clusters displaying different degrees of DNA methylation alteration.

Functional annotation of the DM genes (NIH-DAVID software) identified several enriched Gene Ontology (GO) biological processes (Fisher Exact test). Hypermethylated genes were significantly associated with *nervous system development *(*p*-value = 7e-15), *embryonic development *(*p*-value = 3e-13), *brain development *(*p*-value = 6e-16), and *cell migration *(*p*-value = 4e-4). Hypomethylated genes were significantly associated with *immune response *(*p*-value = 1e-10) and *response to stress *(*p*-value = 8e-16).

**Figure 3:**
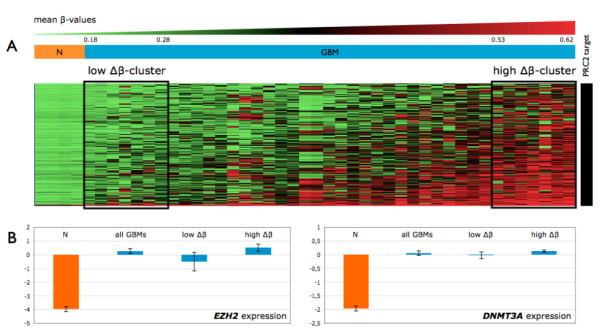
**Analysis of the hypermethylated CpGs located within PRC2-targeted promoters**. (A) Heatmap of the hypermethylated CpGs located within PRC2-targeted promoters. Samples are ranked horizontally as a function of their mean β-values. Two clusters representing extreme methylation changes (Δβ) relative to control samples (N) are framed. (B) *EZH2 *and *DNMT3A *expression level in control samples, GBM samples, the low- and the high-Δβ clusters. The expression values presented are normalized and log-transformed intensities.

Interestingly, 97% of the hypermethylated CpG sites were located within a CpG island, whereas 91% of the abnormally demethylated CpG sites were not located within a CpG island. We compared the frequencies of PRC2 marks in the hypermethylated gene set and in the full array, as previously described by Martinez *et al*. [20]. The hypermethylated gene set was significantly enriched in PRC2 targets (35% vs. 9.5%, Fisher's exact test *p*-value = 2e-16; Figure 2). This suggests that a large proportion of the hypermethylated genes in GBM may have undergone *de novo *DNA methylation mediated by the PRC2 complex. We tested this hypothesis by carrying out unsupervised hierarchical clustering restricted to the hypermethylated CpGs located within PRC2-targeted promoters (Figure 3A). We observed considerable heterogeneity between GBMs and we focused on two groups of seven patients clustered on the basis of the difference between their mean β-value and the one of control brain (Δβ). These groups are named the "low-Δβ" (mean Δβ = 0.15) and "high-Δβ" (mean Δβ = 0.49) groups. We compared the expression levels of genes belonging to the PRC2 complex (*EZH2*, *SUZ12*, *EED*) and *DNMT *genes (*DNMT1*, *DNMT3A *and *DNMT3B*) in control brains, all GBM samples, the low-Δβ cluster and the high-Δβ cluster. Two genes (*EZH2 *and *DNMT3A*) were significantly over-expressed in GBMs relative to control brains (FDR = 0, fold-change = 19 and FDR = 0.003, fold-change = 4, respectively). These two genes were more strongly expressed in the high-Δβ cluster, but no statistically significant difference was found between the levels of expression in the low- and high-Δβ clusters (Figure 3B).

### Correlation analysis

**Figure 4:**
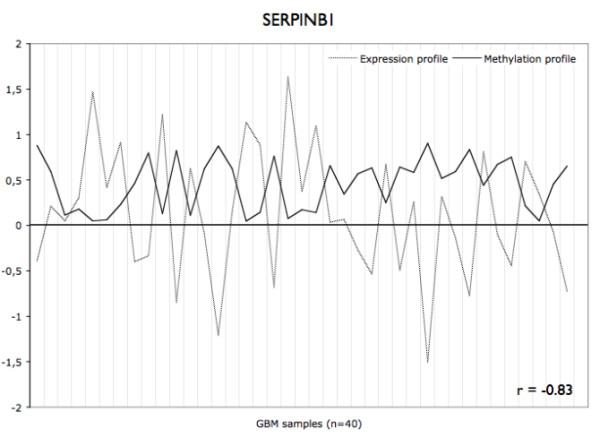
**Expression and methylation profiles for the SERPINB1 gene in GBM patients (n = 40)**. The expression values presented are normalized and log-transformed intensities. The methylation values are β-values.

In total, 421 CpG sites (321 genes) displayed a significant inverse correlation (r < -0.5) between methylation level and the level of expression of the corresponding gene in GBM samples (Additional file 4). Almost 91% of these sites were located within CpG islands. The genes displaying the strongest inverse correlation included four genes related to cancer processes: *SERPINB1 *(Figure 4), which promotes cancer cell motility in invasive oral squamous cell carcinoma [26], *EMP3*, which displays regulation through promoter methylation in gliomas [27], *FABP5*, which mediates EGFR-induced carcinoma cell growth [28], and *CBR1*, which is involved in tumor progression [29,30]. Thirteen genes were DE in GBM vs. control brain, consistent with their promoter methylation status (5 overexpressed genes with a hypomethylated promoter: *B3GNT5*, *FABP7*, ZNF217, *BST2 *and *OAS1*; 8 underexpressed genes with a hypermethylated promoter: *SLC13A5*, *GSTM5*, *ME1*, *UBXD3*, *TSPYL5*, *FAAH*, *C7orf13*, and *C3orf14*).

### Survival analysis

**Figure 5:**
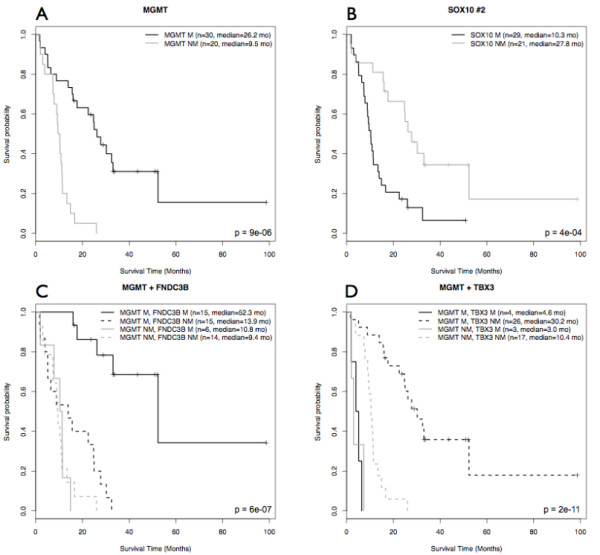
**Kaplan-Meier estimation of overall survival in 50 GBMs treated in accordance with the STUPP protocol**. Patients were assigned to groups according to the methylation status of (A) *MGMT*, (B) *SOX10 *site #2, (C) *MGMT *and *FNDC3B*, and (D) *MGMT *and *TBX3*. M: methylated; NM: non methylated. *P*-values for the difference in OS (log-rank test), size and median survival of each group are also reported. See Table 1 for β-values cut-offs.

Univariate Cox analyses identified 474 CpG sites (419 genes) significantly associated with OS (Additional file 5). These sites had a high predictive power (absolute univariate z score greater than 2) and 26 were inversely correlated. As expected, the methylation status of the five CpG sites located within the *MGMT *promoter was correlated with survival. Sixty CpG sites stratified the patients into two groups (each containing at least five patients) with significantly different OS (Additional file 6). One of these sites is located within the *MGMT *promoter (Table 1 and Figure 5A) and its Illumina probe overlaps the sequence tested by the PyroMark Q96 CpG *MGMT *kit used to validate our data (Additional file 7). For this CpG site, a strong correlation was obtained between the results of the two techniques (r = 0.7). Interestingly, 10 CpG sites (9 genes) had a larger AUC than the *MGMT *CpG (Kruskal-Wallis test *p-*value < 5e-4) (Table 1). For these 10 CpGs no evidence of violation of the proportional hazards assumption was found. The hypermethylation of two of these CpG sites, within the *SOX10 *promoter, was associated with shorter survival (Figure 5B). CpG site #2 methylation level was inversely correlated with the level of *SOX10 *expression (r = -0.75) in GBM samples, and *SOX10 *was significantly underexpressed in GBM (FDR = 0.009, fold-change = 4). This inverse correlation and underexpression in GBM, is entirely consistent with the shorter survival observed for patients displaying *SOX10 *hypermethylation. Four CpG sites remained significantly associated with OS (*p*-value < 0.01) in a Cox multivariate model including *MGMT *promoter methylation status and were therefore identified as potential independent prognostic markers. These sites are located within the promoters of the *FNDC3B*, *TBX3*, *FSD1*, and *DGKI *genes (Figure 5C and 5D and Additional file 8).

**Table 1:** Survival analysis for 50 GBM patients treated with STUPP protocol.

Variable	Univariate Cox Regression			Log Rank Test		Multivariate Cox Regression		
	HR	95% CI	*p*-value	ß cut-off	*p*-value	HR	95% CI	*p*-value
Age (≥ 50 yr vs. < 50 yr)	1.8	0.9 - 3.9	0.1	-	-	-	-	-
Sex (male vs. female)	1.5	0.8 - 2.8	0.3	-	-	-	-	-
KPS (≥ 80 vs. < 80)	1.2	0.6 - 2.3	0.6	-	-	-	-	-

TBX3	113	15.1 - 851.9	4.0E-06	0.45	1.0E-08	0.05	0.01 - 0.2	3.0E-05 (*)
FSD1	18	2.9 - 112	0.002	0.70	3.0E-07	0.2	0.09 - 0.6	0.002 (*)
FNDC3B	0.08	0.01- 0.4	0.002	0.55	7.0E-05	3.1	1.4 - 6.9	0.005
DGKI	77	9.5 - 616.5	4.0E-05	0.45	3.0E-06	0.3	0.1 - 0.7	0.008
FLJ25422	0.05	0.007 - 0.3	0.002	0.70	4.0E-04	2.9	1.2 - 7.2	0.02
SEPP1	0.008	0.003 - 0.2	0.004	0.10	2.0E-04	2.3	1.1 - 4.7	0.02
SOX10 #1	10	1.6 - 67.2	0.01	0.70	1.0E-04	0.4	0.2 - 0.9	0.03
CCND1	31	4.3 - 216	6.0E-04	0.75	2.0E-04	2.3	0.9 - 5.5	0.1
SOX10 #2	12	1.9 - 74.8	0.008	0.80	4.0E-04	0.5	0.2 - 1.2	0.1
ZNFN1A3	0.11	0.02 - 63.8	0.02	0.35	9.0E-04	1.8	0.8 - 4.2	0.2
*MGMT*	*0,18*	*0.04 - 0.8*	*0.02*	*0.10*	*9.0E-06*	-	-	-

Log rank tests were performed between methylated and non-methylated patients. The multivariate analysis includes the methylation status of the tested CpG site and *MGMT*. *MGMT *methylation status was always significantly associated with OS (*p*-value < 0.05) and (*) indicates which CpG site had a lower *p*-value than the one observed for *MGMT *in the multivariate model. (yr = year; HR = Hazard Ratio; CI: Confidence Interval).

## Discussion

In this study, we used array technology for quantitative expression and methylation profiling in a well characterized cohort of newly diagnosed GBM patients. We describe (i) the relationship between DNA methylation pattern and gene expression in GBM and (ii) the association between DNA methylation and clinical outcome in a subgroup of patients given uniform treatment in accordance with the STUPP protocol.

The methylation analysis identified 616 CpG sites DM between GBM and control brain and revealed considerable heterogeneity between GBMs, particularly for hypermethylated CpG sites. Hypo- and hypermethylated CpG sites were preferentially located outside and within CpG islands, respectively. This clearly confirms that cancer cells are characterized by both a loss of methylation in CpG-depleted regions and gains of methylation at CpG islands [4]. Consistent with the findings of Martinez *et al*. [20], the hypermethylated gene set was found to be significantly enriched in PRC2 targets, highlighting the putative role of polycomb group proteins in *de novo *methylation in GBM. However, our data were not entirely consistent with this hypothesis. Indeed, there is no strong methylation pattern among the PRC2 targeted promoters and the changes in expression of the *PRC2 *and *DNMT *genes do not follow the hypermethylation gradient observed between low- and high-Δβ GBM clusters. This suggests that other genes may be linked to polycomb-associated *de novo *methylation.

The integrated analysis of DNA methylation and gene expression showed that DNA methylation only partly regulated gene expression. Indeed, almost a quarter of the DM genes also displayed concordant differential expression (chi-square test *p*-value < 0.01) (Additional file 9) and, in GBM samples, only 3% of the genes displayed an inverse correlation between promoter methylation and expression levels. This finding is consistent with published data for GBM [21]. Moreover, many other well known mechanisms are involved in the regulation of gene expression (e.g. copy number alterations [2,3], transcription factor production and recruitment, histone modifications, micro-RNA expression [31]). Nevertheless, our analysis led to the identification of 13 genes displaying concordant differential methylation and differential expression in GBM and control brain, and whose methylation and expression patterns were anti-correlated. The expression patterns of these genes may therefore be tightly regulated by epigenetic mechanisms, and their in-depth analysis may help us to understand the contribution of DNA methylation to glioblastomagenesis. Most of these genes have already been implicated in cancer-related processes. For example, *ZNF217 *(encoding zinc finger protein 217) is an important oncogene in many cancer types and its overexpression has been implicated in cell immortalization and resistance to chemotherapy [32]. A recent study demonstrated that the ZNF217 protein forms nuclear complexes with several histone-modifying proteins (including EZH2) with synergistic effects in transcriptional repression [33]. Another example is provided by FABP7 (brain fatty acid binding protein 7), which is expressed by the radial glia and involved in glia-guided neuronal migration [34]. This protein has been associated with pure GBM histology, invasion and poor prognosis [35]. Yet another example is provided by *TSPYL5 *(encoding testis-specific Y-like protein), which is a potent tumor suppressor gene and a frequent target of epigenetic silencing in glial tumors and gastric cancers [17,36]. This gene has been shown to play a role in cell growth and resistance to radiation, through regulation of the p21(WAF1/Cip1) and PTEN/AKT pathway [37].

Noushmehr *et al*. [21] described a rare subgroup of GBMs with a CpG Island Methylator Phenotype. These G-CIMP tumors are a subclass of the GBM *proneural *subtype defined by Phillips *et al*. and Verhaak *et al*. [38,39]. They were shown to be associated with secondary and recurrent GBMs, *IDH1 *somatic mutation, younger age at diagnosis and longer survival. Based on the G-CIMP 8-gene signature they describe (*ANKRD43*, *HFE*, *MAL*, *LGALS3*, *FAS-1*, *FAS-2*, *RHO-F*, and *DOCK5*), we identified three G-CIMP-positive tumors in the 55 patients of our cohort. This proportion (5.5%) is similar to that reported in the context of the TCGA (7.6%). We also confirm the association of G-CIMP status with *IDH1 *somatic mutation (Fisher's exact test *p*-value = 2e-4) and younger age at diagnosis (Wilcoxon rank sum test *p*-value = 0.01). However, we were unable to test the association between G-CIMP-positive status and OS, due the low frequency of this phenotype (three patients, two with survival data available).

Survival analysis was performed on a cohort of 50 patients uniformly treated by radiotherapy combined with concomitant and adjuvant temozolomide (STUPP protocol) [40]. To our knowledge, this is the largest uniformly treated GBM cohort ever to be studied over such a large number of CpG loci. As expected, *MGMT *promoter methylation was strongly associated with longer survival, in both the microarray and pyrosequencing approaches. The chosen cutoff point for the β-value (10%) is similar to frequently used values (9%) [41]. For the 27,578 CpG sites tested, *MGMT *methylation status remained one of the most powerful predictors of response to temozolomide-based treatment in GBM. Nevertheless, we have also identified two different types of prognostic markers. The first type stratifies the patients similarly to *MGMT*, but with a higher AUC. There is an association between the methylation level of *MGMT *and *SOX10 *promoters (chi-square test *p*-value < 0.01). The *SOX10 *gene is one such marker, and the hypermethylation of its promoter was associated with shorter survival in our cohort. Interestingly, the SOX10 protein is a marker of oligodendrocytes [42], and the presence of oligodendroglial differentiation areas in GBM has also been associated with longer survival [43]. The second type of prognostic marker (*FNDC3B*, *TBX3*, *DGKI*, and *FSD1*) identifies patients with *MGMT*-methylated tumors not responding to STUPP treatment (Additional file 10). This second group of markers need to be validated on a larger cohort.

## Conclusion

We performed a comprehensive analysis of DNA methylation and gene expression profiles obtained from the same GBM cohort, using array technologies. We identified frequent tumor-specific methylation changes in GBM. Some of these alterations directly affected gene expression, whereas others were significantly associated with the clinical outcome of patients and could be useful for predict the response to standard treatment.

## Authors' contributions

AE and MDT elaborated the experimental design. AE, RB, and FG performed the microarray experiments and AE analyzed data. MDT and MA helped to the statistical analysis. AE wrote the paper. MA and JM helped to draft the manuscript. AE, EV, SS, AH, LR, PM, VQ, and JM discussed the results and commented on the manuscript. JM supervised the study. All authors read and approved the final manuscript.

## Supplementary Material

Additional file 1 **Distribution of the β-values for GBM samples (n = 55) and control brain samples (n = 3)**. Click here for file

Additional file 2 **Genes differentially expressed between GBM and control brain**. Click here for file

Additional file 3 **CpG sites differentially methylated between GBM and control brain**. Click here for file

Additional file 4 **CpG sites displaying an inverse correlation between promoter methylation and expression levels**. Click here for file

Additional file 5 **CpG sites significantly associated with overall survival - univariate Cox regression analysis**. Click here for file

Additional file 6 **CpG sites significantly associated with overall survival - Log rank analysis**. Click here for file

Additional file 7 **MGMT promoter sequence**. Overlap between the sequence tested by the PyroMark Q96 CpG *MGMT *kit and the Illumina probe used to stratify patients (log rank test *p*-value = 9e-06). Numbers indicate positions on the reference genome. Click here for file

Additional file 8 **Kaplan-Meier estimation of overall survival in 50 GBMs treated in accordance with the STUPP protocol**. Patients were assigned to groups according to the methylation status of (A) *SOX10 *site #1, (B) *MGMT *and *FSD1*, and (C) *MGMT *and *DGKI*. M: methylated; NM: non methylated. *P*-values for the difference in OS (log-rank test), size and median survival of each group are also reported. See Table 1 for β-values cut-offs. Click here for file

Additional file 9 **Contingency Table showing differentially expressed and differentially methylated enes**. Click here for file

Additional file 10 **Kaplan Meier estimation of overall survival in 30 GBMs with methylated *MGMT *promoter**. Patient were separated into two groups according to the methylation status of (A) *FNDC3B*, (B) *TBX3*, (C) *DGKI*, and (D) *FSD1*. See Table 1 for β-values cut-offs. Click here for file
