# Supplementary figures and images for: DNA methylation in glioblastoma: impact on gene expression and clinical outcome
Source: BMC Genomics. 2010 Dec 14;11:701. doi: 10.1186/1471-2164-11-701 (PMC3018478; doi:10.1186/1471-2164-11-701)

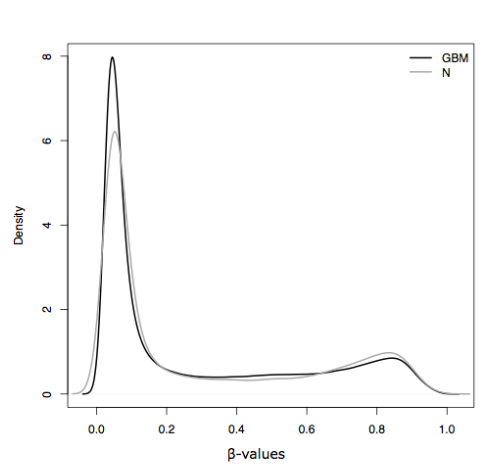

Supplement: Additional file 1 — Distribution of the β-values for GBM samples (n = 55) and control brain samples (n = 3). [file 1471-2164-11-701-S1.TIFF]

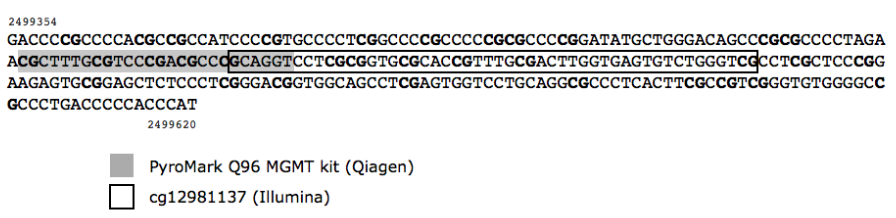

Supplement: Additional file 7 — MGMT promoter sequence. Overlap between the sequence tested by the PyroMark Q96 CpG MGMT kit and the Illumina probe used to stratify patients (log rank test p-value = 9e-06). Numbers indicate positions on the reference genome. [file 1471-2164-11-701-S7.TIFF]

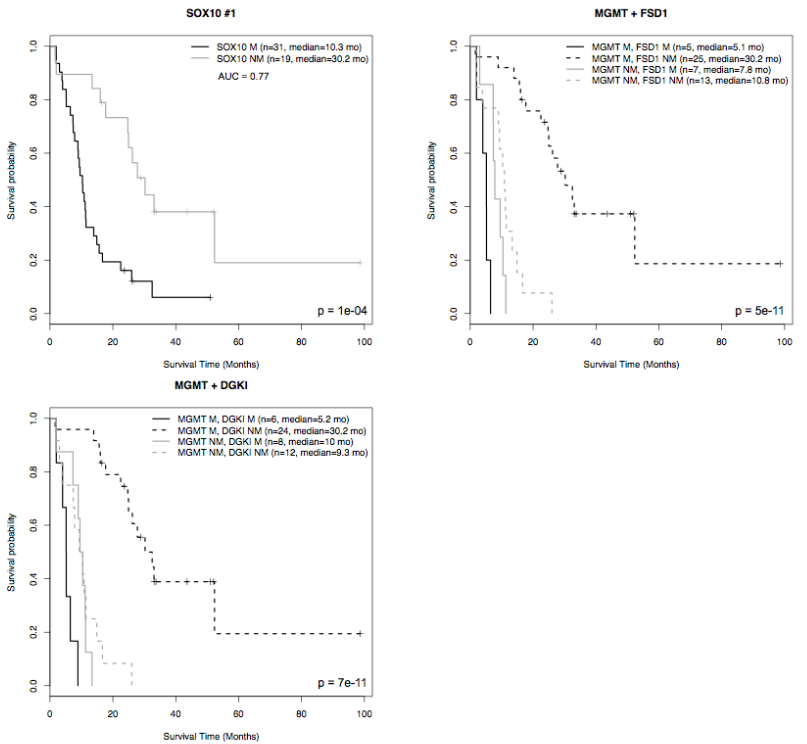

Supplement: Additional file 8 — Kaplan-Meier estimation of overall survival in 50 GBMs treated in accordance with the STUPP protocol. Patients were assigned to groups according to the methylation status of (A) SOX10 site #1, (B) MGMT and FSD1, and (C) MGMT and DGKI. M: methylated; NM: non methylated. P-values for the difference in OS (log-rank test), size and median survival of each group are also reported. See Table 1 for β-values cut-offs. [file 1471-2164-11-701-S8.TIFF]

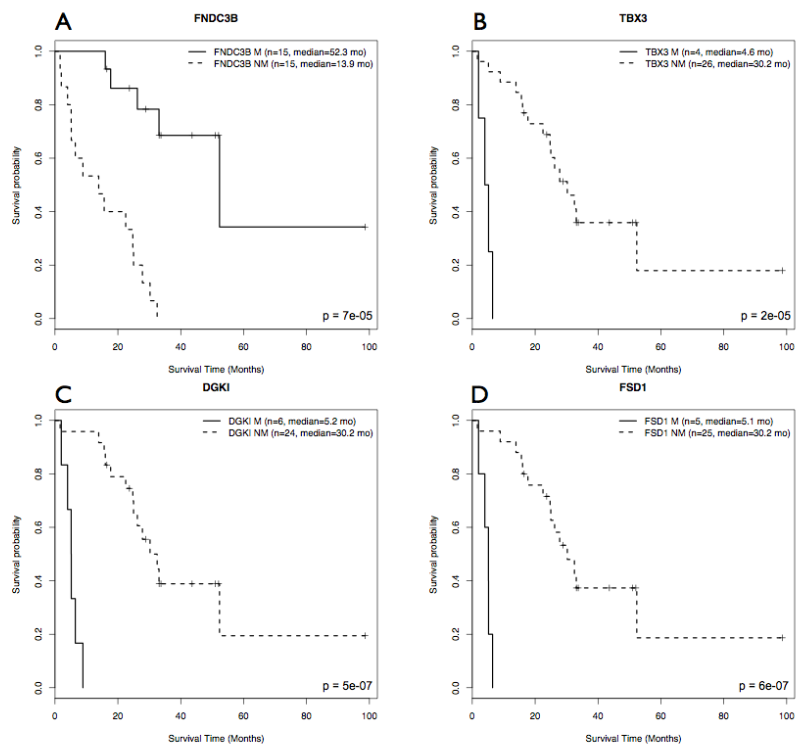

Supplement: Additional file 10 — Kaplan Meier estimation of overall survival in 30 GBMs with methylated MGMT promoter. Patient were separated into two groups according to the methylation status of (A) FNDC3B, (B) TBX3, (C) DGKI, and (D) FSD1. See Table 1 for β-values cut-offs. [file 1471-2164-11-701-S10.TIFF]
